# A hyperacute immune map of ischaemic stroke patients reveals alterations to circulating innate and adaptive cells

**DOI:** 10.1111/cei.13551

**Published:** 2020-12-09

**Authors:** S. Krishnan, C. O’Boyle, C. J. Smith, S. Hulme, S. M. Allan, J. R. Grainger, C. B. Lawrence

**Affiliations:** ^1^ Geoffrey Jefferson Brain Research Centre Faculty of Biology, Medicine and Health Manchester Academic Health Science Centre University of Manchester Manchester UK; ^2^ Lydia Becker Institute of Immunology and Inflammation Faculty of Biology, Medicine and Health Manchester Academic Health Science Centre University of Manchester Manchester UK; ^3^ Division of Infection, Immunity and Respiratory Medicine School of Biological Sciences Faculty of Biology, Medicine and Health University of Manchester Manchester UK; ^4^ Division of Neuroscience and Experimental Psychology School of Biological Sciences Faculty of Biology, Medicine and Health University of Manchester Manchester UK; ^5^ Division of Cardiovascular Sciences University of Manchester Manchester Academic Health Science Centre Salford Royal NHS Foundation Trust Salford UK; ^6^ Manchester Centre for Clinical Neurosciences Salford Royal NHS Foundation Trust Salford UK

**Keywords:** clinical study, ischaemic stroke, neuroimmunology, stroke immunophenotypes, systemic immunity

## Abstract

Systemic immune changes following ischaemic stroke are associated with increased susceptibility to infection and poor patient outcome due to their role in exacerbating the ischaemic injury and long‐term disability. Alterations to the abundance or function of almost all components of the immune system post‐stroke have been identified, including lymphocytes, monocytes and granulocytes. However, subsequent infections have often confounded the identification of stroke‐specific effects. Global understanding of very early changes to systemic immunity is critical to identify immune targets to improve clinical outcome. To this end, we performed a small, prospective, observational study in stroke patients with immunophenotyping at a hyperacute time point (< 3 h) to explore early changes to circulating immune cells. We report, for the first time, decreased frequencies of type 1 conventional dendritic cells (cDC1), haematopoietic stem and progenitor cells (HSPCs), unswitched memory B cells and terminally differentiated effector memory T cells re‐expressing CD45RA (TEMRA). We also observed concomitant alterations to human leucocyte antigen D‐related (HLA‐DR), CD64 and CD14 expression in distinct myeloid subsets and a rapid activation of CD4^+^ T cells based on CD69 expression. The CD69^+^CD4^+^ T cell phenotype inversely correlated with stroke severity and was associated with naive and central memory T (TCM) cells. Our findings highlight early changes in both the innate and adaptive immune compartments for further investigation as they could have implications the development of post‐stroke infection and poorer patient outcomes.

## Graphical abstract

Stroke affects millions of people across the globe and infections following a stroke often lead to death or disability in patients. Looking rapidly after a stroke in patients, we identify early alterations to the innate and adaptive cells in circulation. Our findings highlight novel changes to dendritic cells, monocytes, haematopoietic stem and progenitor cells, B and T cells to be further investigated as they could have implications the development of post‐stroke infection and poorer patient outcomes.

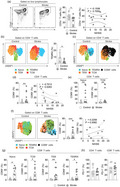

## Introduction

Stroke (cerebral ischaemia) is the leading cause of adult disability and a major cause of death worldwide [1, 2]. In addition to initial neurological impairments, such as dysphagia, stroke can rapidly exacerbate the risk of bacterial pneumonia which is independently associated with increased mortality and worse functional outcome in survivors [3, 4, 5, 6]. Bacterial pneumonia could, in part, result from a pneumonitis with an inflammatory component [7, 8]. Together with reduced motor control (e.g. contralateral respiratory muscle/diaphragmatic weakness contributing to hypoventilation) [9] and increased vagal drive [10], treatment in a high infection risk environment could also exacerbate an environment for the development of bacterial pneumonia. The ineffectiveness of candidate primary interventions such as prophylactic β‐blockers and antibiotic therapy in preventing stroke‐associated pneumonia (SAP) has necessitated a clearer understanding of the systemic immune landscape in stroke, to identify mechanisms underlying early complications and new therapeutic approaches to improve patient outcome [11, 12, 13]. As most infections, particularly SAP, manifest within the first 72 h of stroke onset [14], novel strategies to prevent SAP need to be applied as soon as possible after stroke occurrence. Defining changes in the circulating immune cell compartment very early (within hours) after stroke onset, prior to manifestation of infections, is therefore critical.

Cerebral ischaemia drives local microglial activation in tandem with peripheral immune cell (e.g. monocytes, neutrophils, B cells and T cells) recruitment into the brain, as well as the production and release of cytokines and chemokines [15]. The ensuing inflammation is adversely involved in the evolution of stroke pathology and through its impact on systemic immunity leads to an increased risk of infection by impairing anti‐microbial function. The mechanisms underlying systemic immune changes after stroke are not fully understood, but increased catecholamine release by the sympathetic nervous system, glucocorticoids and changes in cholinergic output are proposed [4, 16, 17, 18].

There exists a wealth of literature implicating alterations to the abundance or functional capacity of almost all immune cells, including T cell subsets [19, 20, 21, 22, 23, 24, 25], B cells [26, 27, 28], invariant natural killer T (iNK T) cells [29], mononuclear phagocytes [30, 31, 32, 33] and neutrophils [34, 35, 36] following acute ischaemic stroke. Commonly reported immunophenotypes are lymphopenia [37], elevated neutrophil–lymphocyte ratios [38, 39], decreased human leucocyte antigen D‐related (HLA‐DR) expression on monocytes [31, 32] and hypogammaglobulinaemia [26, 40], as well as impaired production of reactive oxygen species by neutrophils [34, 36]. In many of these studies, alterations to individual populations or specific arms of the immune system (i.e. innate or adaptive) were characterized and a more global immunophenotyping approach was not taken. Moreover, acute changes to systemic immunity have typically been characterized within 12 or 24 h of disease onset [3, 29, 34, 41, 42, 43, 44]. To date, the earliest point at which alterations to immunity have been documented is 3 h following stroke onset, predominantly focusing upon monocytes, their phenotype and relationship with clinical outcome [37]. Thus, the characteristic changes exhibited between myeloid and lymphoid immune populations following ischaemic stroke have not been concurrently documented in the same patient cohort in the hyperacute (< 3 h) phase.

To this end, we performed a small, prospective, observational immunophenotyping study where we explored the profile of forty immune parameters across three panels on whole blood cells from ischaemic stroke patients at a median time of 168 min following stroke symptom onset and in controls of similar age and sex distribution. Our data identify for the first‐time decreased frequencies of a specific subset of dendritic cells (DC) (type I conventional dendritic cells, cDC1), altered expression of HLA‐DR, CD64 and CD14 in distinct myeloid populations and alterations to haematopoietic stem and progenitor cells (HSPC). We also show a previously undescribed modulation of memory B cells as well as terminally differentiated effector memory T cells re‐expressing CD45RA (TEMRA). Based on CD69^+^ expression, we also observed a rapid activation of naive and central memory T (TCM) CD4^+^ T cells, the latter of which are capable of migration to lymphoid organs and orchestrate antigen recall responses [45]. The frequency of CD69^+^CD4^+^ T cells inversely correlated with stroke severity. Our findings highlight unappreciated early changes in both the innate and adaptive immune compartments to be validated in larger cohorts of patients, and provide valuable hypothesis generation regarding how diverse immune cell populations could independently and differentially change following stroke.

## Materials and methods

### Participants, study design and sample acquisition

Samples in this study were part of the flow cytometry substudy and obtained from participants recruited as part of the Subcutaneous Interleukin‐1 Receptor Antagonist (IL‐1Ra) in Stroke Study [46]. This was a Phase II clinical trial conducted at Salford Royal NHS Foundation Trust (SRFT) and received ethical approval from the Greater Manchester South NHS Research Ethics Committee, UK Medicines and Healthcare Products Regulatory Agency (MHRA) and SRFT Research and Development (Ethics ref.: 13/NW/0460; EudraCT no: 2013‐001757‐28; Sponsor Ref no.: 2013/066st). In brief, patients aged over 18 years with a diagnosis of ischaemic stroke < 5 h of symptom onset presenting at the Manchester Centre for Clinical Neurosciences (MCCN) Hyper Acute Stroke Unit (HASU) were recruited as previously described [46]. Approval was obtained to draw a blood sample immediately on admission (median time to sample was 168 min), prior to thrombolysis (when eligible), randomization to treatment and receipt of consent for participation in the Phase II trial. The National Institutes of Health Stroke Scale (NIHSS) was used to assess stroke severity at admission; investigational treatment was administered < 6 h within symptom onset. Community‐dwelling control participants with similar age and sex distribution to the patients and with no prior history of transient ischaemic attack or stroke, or infection treated with antibiotics within the preceding 6 weeks and capable of informed consent were also recruited. The demographics and baseline characteristics of the cohort are summarized in Table 1.

**Table 1:** Summary demographics of sampled population

	Control (15)	Stroke (13)
Age (years)	70 (65–76)	75 (65–81)
Sex		
Male	9 (60%)	8 (61·5%)
Female	6 (40%)	5 (38·5%)
Co‐morbidities		
Smoking		
Smoker	0/15 (0%)	3/11 (27·3%)
Ex‐smoker	6/15 (40%)	2/11 (18·2%)
Non–smoker	9/15 (60%)	6/11 (54·5%)
Diabetes	5/14 (35·7%)	2/12 (16·7%)
Hypertension	7/14 (50%)	5/12 (41·7%)
PVD	5/14 (35·7%)	0/11 (0%)
CAD	0/14 (0%)	3/12 (25%)
AF	0/14 (0%)	1/11 (9·1%)
COPD	0/14 (0%)	1/10 (10%)
Previous stroke/TIA	0/14 (0%)	3/13 (23·1%)
Stroke severity (NIHSS)		
Mild (0–4)	–	1/13 (7·7%)
Moderate (5–15)	–	7/13 (53·8%)
Severe (> 16)	–	5/13 (38·5%)
Thrombolysis	–	11/13 (84·6%)
Infection		
At sample/admission	0/15	1/13 (7·7%)
Post stroke	–	4/13 (30·8%)
Pneumonia	–	3/13 (23·1%)
UTI	–	1/13 (7·7%)
Acute phase response		
CRP	–	2·21 (0·55–9·84)^2^
IL–6	–	6·56 (0·58–8·07)^2^
vWF	–	1·14 (0·97–2·55)^2^
Differential whole blood counts		
WBC	–	7·60 (6·35–8·75)
Lymphocytes	–	1·60 (1·35–2·10)
Neutrophils	–	4·60 (3·60–6·15)
Monocytes	–	0·40 (0·40–0·65)
Eosinophils	–	0·20 (0·10–0·30)
Basophils	–	0·10 (0·05–0·10)
Neutrophil–lymphocyte ratio	–	2·75 (2·12–3·96)

Data are median (interquartile range)^m^ where ^m^ is the number of missing data points.

*n/N* (%), where *N* is the total number of observations with available data.

AF = atrial fibrillation; CAD = coronary artery disease; COPD = chronic obstructive pulmonary disease; TIA = transient ischaemic attack; PVD = peripheral vascular disease; NIHSS = National Institutes of Health Stroke Scale; UTI = urinary tract infection; CRP = C‐reactive protein; IL‐6 = interleukin‐6; vWF = von Willebrand factor; WBC = white blood cells.

### Cell isolation from blood

Briefly, 3 ml of venous blood collected in ethylenediamine tetraacetic acid (EDTA) tubes (Sarstedt, Leicester, UK) was washed in phosphate‐buffered saline (PBS) and resuspended in 9 ml of sterile water (Hyclone, Cramlington, UK) for 10 sec at room temperature twice. Cell suspensions were washed, resuspended in PBS and counted before staining.

### Flow cytometry

Single‐cell suspensions of blood (~3 × 10^6^–5 × 10^6^ total cells) were incubated in PBS for 15 min at 4°C in the dark with the Zombie UV™ or Zombie Aqua™ Fixable Viability Kit (BioLegend, London, UK) as appropriate and immunoglobulin (Ig)G from human serum (Sigma Aldrich, Gillingham, UK). Cells were washed in PBS and stained for a further 15 min in three cocktails of fluorochrome‐conjugated antibodies to identify myeloid, B or T cell subsets. Our immunophenotyping panels were adapted from Haniffa *et al*. [47], Tsang *et al*. [48], Thome *et al*. [49] and Thome *et al*. [50], which are summarized in Table 2. Cells were fixed in 2% paraformaldehyde (Sigma Aldrich) at room temperature for 10 min, washed and resuspended in PBS prior to acquisition. Samples were acquired on an LSR Fortessa using facsdiva version 8 software (BD Biosciences) and, typically, all cells in the sample were collected. Data were analysed using FlowJo software (Treestar, Inc., Ashland, OR, USA). We identified all immune cells based on their expression of CD45 and the lineages were determined based on the indicated markers:

**Table 2:** Staining panels used in immunophenotyping stroke patients

Colour	Antibody	Clone	Supplier
Myeloid panel			
FITC	CD3	UCHT1	Biolegend
FITC	CD19	HIB19	Biolegend
FITC	CD20	2H7	Biolegend
FITC	CD56	MEM‐188	Biolegend
FITC	CD66b	G10F5	Biolegend
FITC	CD15	W6D3	Biolegend
PerCP‐Cy5.5	CX3CR1	2A9‐1	Biolegend
APC	CD11c	B‐ly6	BD Pharmingen
AF700	CD14	M5E2	Biolegend
APC‐Cy7	CD16	3G8	Biolegend
BV421	CD192 (CCR2)	K036C2	Biolegend
BV510	Zombie Aqua		Biolegend
BV605	CD34	581	Biolegend
BV650	HLA‐DR	L243	Biolegend
BV785	CD123	6H6	Biolegend
PE	CD141 (BDCA‐3)	AD5‐14H12	Miltenyi Biotec
PE‐Cy7	CD1c	L161	Biolegend
PE‐Dazzle™ 594	CD64	10.1	Biolegend
BUV395	CD45	HI30	BD Biosciences
B cell panel			
FITC	CD3	UCHT1	Biolegend
PerCP‐Cy5.5	CD23	EBVCS‐5	Biolegend
APC	CD27	M‐T271	Biolegend
AF700	CD20	2H7	Biolegend
APC‐Cy7	IgM	MHM‐88	Biolegend
BV421	IgD	IA6‐2	Biolegend
BV510	Zombie Aqua		Biolegend
BV650	CD38	HB‐7	Biolegend
BV711	CD19	HIB19	Biolegend
PE	CD10	HI10a	Biolegend
PE‐Cy7	CD1c	L161	Biolegend
PE‐Dazzle™ 594	CD45R	RA3‐6B2	Biolegend
T cell panel			
FITC	Vδ2	B6	Biolegend
PerCP‐Cy5.5	CD4	OKT4	Biolegend
APC	CD69	FN50	Biolegend
AF700	CD3	UCHT1	Biolegend
APC‐Cy7	CCR7	G043H7	Biolegend
BV421	FoxP3	206D	Biolegend
BV510	CD25	M‐A251	Biolegend
BV605	CD45RA	HI100	Biolegend
BV650	CD8	SK1	Biolegend
BV711	CD45	HI30	Biolegend
BV785	CD127	A019D5	Biolegend
UV	Zombie UV		Biolegend
PE	PD1	EH12.2H7	Biolegend
PE‐CF594	TCRγδ	B1	Biolegend

FITC = fluorescein isothiocyanate; PerCp‐Cy.5·5 = peridinin chlorophyll‐cyanin 5.5; APC = allophycocyanin; PE = phycoerythrin; TCR = T cell receptor; PD1 = programmed cell death 1; FoxP3 = forkhead box protein 3; Ig = immunoglobulin; HLA‐DR = human leucocyte antigen D‐related; AF700 = Alexa Fluor 700; BV = brilliant violet.

#### Myeloid cells

Myeloid cells were as follows: neutrophils: SSC^hi^CD66b^+^CD16^+^; basophils: SSC^low^HLA‐DR^−^CD123^+^; classical monocytes: Lin (CD3ε, CD19, CD20, CD15, CD56, CD66b)^−^ HLA‐DR^+^CD14^++^CD16^−^ ; intermediate monocytes: Lin^−^HLA‐DR^+^CD14^++^CD16^+^; non‐classical monocytes: Lin^−^HLA‐DR^+^CD14^+^CD16^++^; conventional DC1: Lin^−^HLA‐DR^+^CD14^−^CD16^−^CCR2^+^CD141^+^; conventional DC2: Lin^−^HLA‐DR^+^CD14^−^CD16^—^CCR2^+^CD1c^+^; plasmacytoid DC: Lin^−^HLA‐DR^+^CD14^−^CD16^−^CCR2^+^CD123^+^; and haematopoietic stem and progenitor cells: Lin (CD3ε, CD19, CD20, CD15, CD56, CD66b, HLA‐DR, CD123, CD14, CD16, CD11c, CD64)^−^CD34^+^.

#### B cells

B cells were as follows: CD3ε^−^CD19^+^CD20^+^; plasmablasts: (B cells) CD27^high^CD38^high^; unswitched memory: (B cells) CD27^+^IgD^+^; switched memory: (B cells) CD27^+^IgD^−^; naive: (B cells) CD27^−^IgD^+^; and double‐negative (B cells) CD27^−^IgD^−^.

#### T cells

T cells were as follows: CD45^+^CD3ε^+^; γδ T cells: (T cells) T cell receptor (TCR)‐γδ^+^Vδ^+/−^; CD4^+^ T cells: (T cells) TCR‐γδ^−^CD4^+^; CD8^+^ T cells: (T cells) TCR‐γδ^−^CD8^+^; among CD4 and CD8 T cells, we identified TEM: CCR7^−^CD45RA^−^; TCM: CCR7^+^CD45RA^−^; naive: CCR7^+^CD45RA^+^; and TEMRA: CCR7^−^CD45RA^+^.

#### Dimensional reduction of flow cytometry data

Flow cytometry data were gated to exclude debris, dead cells and doublets leaving behind live cells for Boolean gating and high‐dimensional analyses. We employed a combination of expert guided manual gating and dimensional reduction using the uniform manifold approximation and projection (UMAP) algorithm, which we implemented in FlowJo. We adopted UMAP over t‐distributed stochastic neighbor embedding (t‐SNE), as it faithfully visualizes cell clusters in the high‐dimensional space following dimensional reduction with a shorter run time [51, 52]. UMAP plots were generated separately for individual samples, such that either the same number of cells or all available cells were sampled to avoid artefacts due to insufficient representation of cells in the sampled gate. Immune cells manually identified based on previously described characteristics were projected on the UMAP to generate a global immune map.

### Statistical analyses

Statistical analyses were performed using Prism version 7/8 software (GraphPad, San Diego, CA, USA) and data are presented as median with individual data points. Statistical comparisons were performed using a Mann–Whitney *U*‐test and correlations using Spearman’s ranked coefficient correlation test. Following sample processing, surface staining and data acquisition, if insufficient cells were present in individual samples for specific panels they were excluded from analyses and appropriate samples numbers are indicated in the Figure legends. One control and stroke patient were excluded from all analyses as indicated in the results and a further three controls and two stroke patients lacked data for up to two panels.

## Results

### Study cohort

We established a research protocol to rapidly sample peripheral blood from ischaemic stroke patients upon admission to the HASU at the MCCN for deep immune profiling as well as assaying acute‐phase proteins. In total, we recruited 13 patients who were stratified for stroke severity according to the NIHSS, of which we analysed 12 samples. We also recruited 16 control participants of similar age and sex distribution in our study (Table 1), of which we analysed 15 samples. One control was excluded from all analyses due to a previous diagnosis of chronic lymphoid leukaemia, while one stroke patient was excluded, as sample processing yielded no viable cells for analysis. Our cohort of stroke patients was 62% male, with a median age of 75 [interquartile range (IQR) = 65–81] years whose characteristics are summarized in Table 1. The median time to blood sampling following stroke symptom onset was 168 min, at which point 85% of the patients were also thrombolysed after blood draw was obtained.

### Stroke alters the composition and phenotype of circulating myeloid cells in the hyperacute phase

**Fig. 1:**
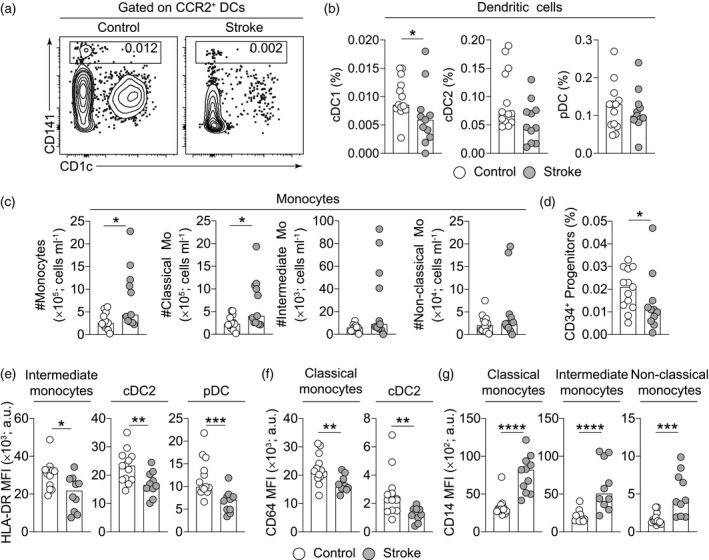
Stroke drives alterations to phenotype of circulating monocyte and dendritic cell subsets in the hyperacute phase. (a) Representative contour plots showing conventional dendritic cells (cDC1s) in the peripheral blood of control and stroke patients as a frequency of live cells. (b) Frequency amongst live cells of conventional dendritic type 1 (cDC1), type 2 (cDC2) and plasmacytoid DCs (pDC) in control (*n* = 13) and stroke patients (*n* = 11). (c) Absolute numbers of monocytes and their subsets in control (*n* = 13) and stroke patients (*n* = 11). (d) Frequency amongst live cells of CD34^+^ haematopoietic stem and progenitor cells in control (*n* = 13) and stroke patients (*n* = 11). (e–g) Quantification of the median fluorescence intensities (MFI) of human leucocyte antigen D‐related (HLA‐DR) (e), CD64 (f) and CD14 (g) in the indicated populations in controls (*n* = 13) and stroke patients (*n* = 10). Data are presented as bars showing median values and dots representing individual data points and statistical comparisons were performed using a Mann Whitney *U* test, ****P < 0·0001, ***P < 0·001, **P < 0·01, *P < 0·05.

To characterize the heterogeneity of myeloid cells, we gated them to identify granulocyte, monocyte and dendritic cell subsets using well‐established markers [53, 54, 55] (Supporting information, Fig. S1a). We identified all DCs based on their expression of CCR2 (Supporting information, Fig. S2a) and divided them into functionally distinct populations based on surface marker expression, notably types 1 and 2 conventional DC (cDC1 and cDC2) and plasmacytoid DC (pDC) [47, 56]. The quantification of manually gated populations revealed a modest decrease in the frequency of cDC1s (Fig. 1a,b), while the frequencies of other myeloid populations remained unaltered (Supporting information, Fig. S2b–d). The frequencies of cDC1s post‐stroke, however, did not correlate with the severity of stroke (Supporting information, Fig. S2e). Enumerating the numbers of immune populations revealed a modest increase in monocytes, particularly classical monocytes (Fig. 1c). We also identified HSPCs based on CD34 expression and the lack of canonical lineage markers and observed a modest decrease in the frequencies of CD34^+^ HSPCs (Fig. 1D and Supporting information, Fig. S1b) that could be suggestive of alterations in haematopoietic output or potential.

Alterations to the surface phenotype myeloid populations have been linked to impairments in the ability to generate appropriate immune responses following infection. For example, the expression of HLA‐DR has been shown to be critical for presenting processed antigens across a variety of cell lineages [57, 58, 59], while the FcγR has been shown to be indispensable for antigen uptake by cDC2s [60, 61]. Therefore, we next investigated the surface phenotype of myeloid cells which highlighted HLA‐DR, CD64 and CD14 as markers that were altered hyperacutely (Fig. 1e–g). Indeed, we identified a modest down‐regulation of HLA‐DR on intermediate monocytes, cDC2s and pDCs post‐stroke, correlating with age but not stroke severity for cDC2s (Fig. 1e and Supporting information, S2f). We also observed a significant down‐regulation of CD64 (FcγRI; high‐affinity Fc receptor for monomeric IgG) on classical monocytes and cDC2s (Fig. 1f). Furthermore, we identified increased CD14 (Toll‐like receptor 4 signalling co‐receptor) expression across all monocyte subsets (Fig. 1g). CD14 up‐regulation has been linked to the acquisition of a tolerance to endotoxin challenge [62]. Taken together, our data could imply rapid alterations in antigen presentation, phagocytic capacity and the ability to secrete cytokines in response to infections following ischaemic stroke.

### Stroke rapidly decreases the frequency of unswitched memory B cells in circulation

**Fig. 2:**
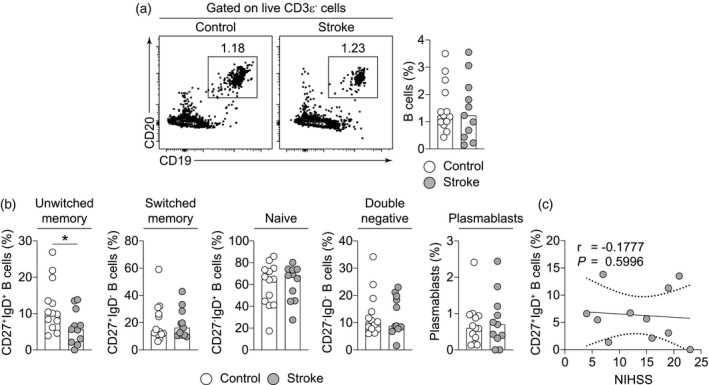
Stroke rapidly decreases the frequency of unswitched memory B cells in circulation. (a) Representative contour plots and quantification of B cells in the peripheral blood of control and stroke patients as a frequency of live lymphocytes. (a) Frequencies of unswitched memory [CD27^+^immunoglobulin (Ig)D^+^], switched memory (CD27^+^IgD^−^), naive (CD27^−^IgD^+^), double‐negative (CD27^−^IgD^−^) B cells and plasmablasts (CD27^+^CD38^+^) among total B cells in control (*n* = 13) and stroke patients (*n* = 11). (c) Correlation plot of unswitched B cell frequencies with stroke severity [National Institutes of Health Stroke Scale (NIHSS)]. Data are presented as bars showing median values and dots representing individual data points. In correlations, the regression line and standard error are shown. Statistical comparisons were performed using a Mann‐Whitney *U*‐test and correlations using Spearman’s ranked coefficient correlation test; *P < 0·05.

Stroke has been shown to drive the loss of B cells in experimental stroke [26], and they have been linked to cognitive decline post‐stroke due to their recruitment to the ischaemic brain in patients [27]. To understand the early impact of cerebral ischaemia on B cells, we identified B cells in whole blood of stroke patients and observed that at a hyperacute time‐point, frequencies of B cells were largely unaltered (Fig. 2a). Emerging research has highlighted impairments in antibody‐mediated immunity as a key driver of post‐stroke infections and the critical role that B cell subsets play [26, 40, 63]. We next identified memory B cell subsets based on their expression of IgD and CD27 [48], which revealed a decrease in the frequency of unswitched memory B cells (Fig. 2b), but this did not correlate with the stroke severity (Fig. 2c). Although plasmablasts and other memory B cell populations remained unaffected (Fig. 2b), a larger cohort might be required to identify changes to the pool of memory B cells. Given that unswitched B cells can rapidly mount IgM‐driven anti‐microbial responses and enter the germinal centre reaction [64, 65, 66, 67], our observations suggest that stroke rapidly drives deficiencies in humoral immunity that could modulate infection susceptibility in patients.

### Stroke rapidly alters the phenotype of the memory T cell compartment in circulation

**Fig. 3:**
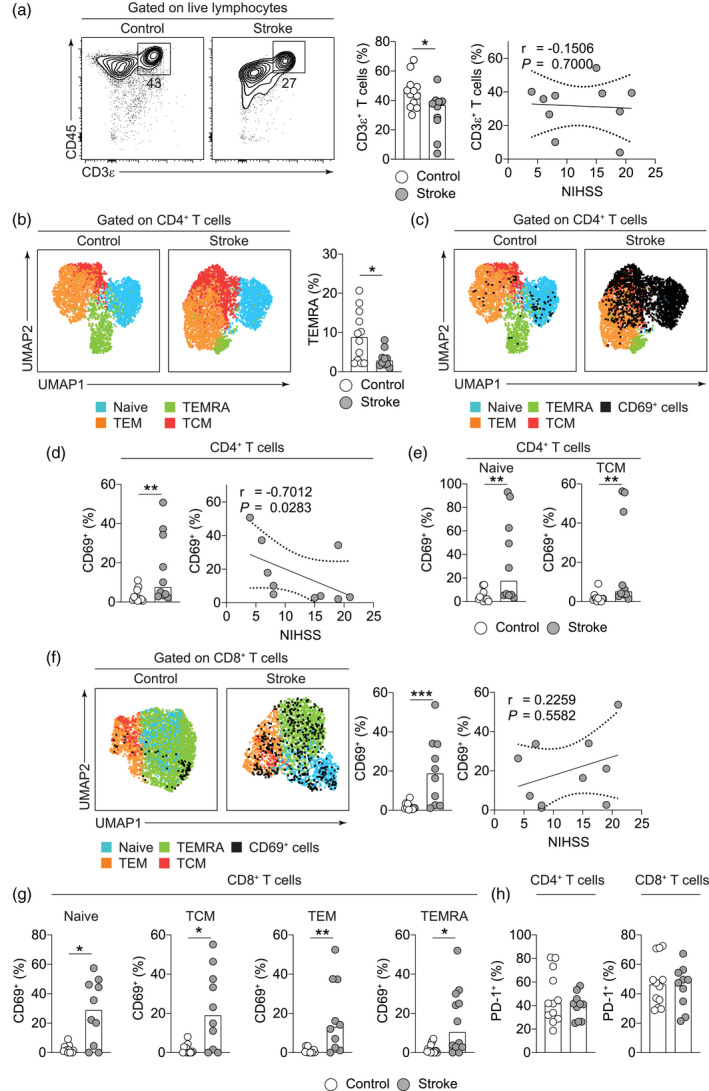
Stroke rapidly alters the phenotype of the T cell compartment in circulation. (a) Representative contour plots of all T cells in the peripheral blood of and stroke patients, quantification of frequencies as a percentage of live lymphocytes and their correlation with stroke severity [National Institutes of Health Stroke Scale (NIHSS)]. (b) Representative uniform manifold approximation and projection (UMAP) plots of CD4^+^ T cells showing memory subsets and quantification of frequency of terminally differentiated effector memory T cells re‐expressing CD45RA (TEMRA) cells as a percentage of CD4^+^ T cells. (c) Representative UMAP plots of CD4^+^ T cells showing an overlay of CD69^+^ cells on memory CD4^+^ T cell subsets. (d) Frequencies of CD69^+^ cells among CD4^+^ T cells in control and stroke patients and their correlation with stroke severity. (e) Frequencies of CD69^+^ cells among CD4^+^ naive and central memory T cells (TCM) cells in control and stroke patients. (f) Representative UMAP plots of CD8^+^ T cells showing an overlay of CD69^+^ cells on memory CD8^+^ T cell subsets, quantification of the proportion of CD69^+^CD8^+^ T cells and their correlation with stroke severity (NIHSS). (g) Quantification of the proportion of CD69^+^ cells among memory CD8^+^ T cell subsets in controls and stroke patients. (h) Frequency of programmed cell death (PD)‐1^+^CD4^+^ and CD8^+^ T cells in controls and stroke patients. Data are presented as bars showing median values and dots represent individual data points for control (*n* = 12) and stroke patients (*n* = 10). In correlations, the regression line and standard error are shown. Statistical comparisons were performed using a Mann–Whitney *U*‐test and correlations using Spearman’s ranked coefficient correlation test; ***P < 0·001, **P < 0·01, *P < 0·05.

Clinical studies have identified the loss of T cells in circulation [25, 68, 69] as well as long‐term functional alterations [20] as a characteristic of stroke‐induced immunosuppression. We first identified CD3ε^+^ T cells in the peripheral blood of controls and stroke patients. In accordance with previous studies, we identified a decrease in the frequency of T cells, but this did not correlate with stroke severity (Fig. 3a). Due to the segregation of functions between TCR‐αβ^+^ and TCR‐γδ^+^ T cells and their subsets [70, 71, 72], as well as the role of IL‐17^+^ γδ T cells in exacerbating ischaemic injury [22, 73], we characterized T cell subsets based on TCR as well as CD4 and CD8 expression. However, stroke did not impact the frequencies of CD4, CD8 or TCR‐γδ^+^ T cell subsets at a hyperacute time‐point (Supporting information, Fig. S3a).

To investigate the activation and exhaustion characteristics acquired by T cell subsets following stroke, we implemented the UMAP algorithm [51, 52] on CD4^+^ and CD8^+^ T cells and projected memory T cell subsets identified by their expression of CCR7 and CD45RA [49] on the dimensional reduction, and observed a decrease in TEMRA cells among CD4^+^ but not CD8^+^ cells (Fig. 3b and Supporting information, S3b). However, we observed a high degree of variability in the frequencies of memory CD8^+^ T cell subsets amongst patients, potentially ascribed to co‐morbidities that were not matched with controls (Supporting information, Fig. S3b). Both the CD4^+^ and CD8^+^ compartments were rapidly activated by stroke, evidenced by the increased proportion of cells expressing CD69 (Fig. 3c–f), a marker of early T cell activation [74]. Increased frequencies of CD69^+^ T cells inversely correlated with stroke severity only in the CD4^+^ compartment (Fig. 3d). The increase in CD69^+^CD4^+^ cells was restricted to naive and central memory T cells (TCM), which also inversely correlated with stroke severity (Fig. 3e and Supporting information, S3c), while all memory subsets up‐regulated CD69 in the CD8^+^ compartment (Fig. 3g). Finally, examining programmed cell death (PD)‐1^+^ T cells within the CD4^+^ and CD8^+^ compartments as a measure of T cell exhaustion [75, 76] revealed no alterations in PD‐1^+^ cells within either compartment at a hyperacute time point (Fig. 3h). Combined, our data outline how stroke swiftly alters the composition and phenotype of myeloid and lymphoid cells in circulation and could have implications for antigen presentation and circuits of humoral immunity as well as memory T cell responses.

## Discussion

Infectious complications following ischaemic stroke present a significant barrier to recovery and are thought to be driven by alterations to the systemic immune landscape. Here, we present a hyperacute map of circulating immune cells in ischaemic stroke patients where we show decreased frequencies of cDC1s, HSPCs, unswitched memory B cells and TEMRA cells. We also identify concomitant alterations in the expression of HLA‐DR, CD64 and CD14 in distinct myeloid subsets and a rapid activation of CD4^+^ T cells based on CD69 expression. Interestingly, this CD69^+^CD4^+^ T cell phenotype inversely correlated with stroke severity and was associated with naive and TCM cells.

The vast majority of CD4^+^ or CD8^+^ T cells have been shown to be CD69^−^ in circulation [77] and the up‐regulation of CD69 is thought to be a marker of early T cell activation, probably in response to the cytokine milieu and TCR engagement, which regulates cytokine production [74]. Although we observe an activation of T cell subsets post‐stroke, the pathways driving their activation remain to be determined. Similarly, while we also implicate CD4^+^ TEMRA cells in the hyperacute immune response following stroke, further studies in larger patient cohorts are essential to determine their functional consequence and temporality, particularly in the context of long‐term cognitive decline, as increased CD8^+^ TEMRA cells in circulation have been recently identified as an immunophenotype in patients with mild cognitive impairment or Alzheimer’s disease [78]. Given that TEMRA cells possess the capacity to migrate to peripheral tissues and take up residence [79], mechanistic studies are critical to determine whether the decreased frequencies observed could be attributed to their migration or loss via cell death. Studies have documented increased CD69^+^ T cells in the palatine tonsils and cervical lymph nodes of stroke patients 76 h following stroke onset [80], and that activated T cells reactive to myelin oligodendrocyte glycoprotein accumulate in the brain following experimental stroke, implicating activated T cells in driving autoimmunity post‐stroke [81, 82]. Conversely, an exhausted T cell phenotype characterized by an increased frequency of PD‐1^+^CD4^+^ T cells has also been observed in patients 48 h post‐stroke [83]. Given the diverse T cell phenotypes and their pleiotropic roles post‐stroke, it becomes crucial to dissect mechanisms that control immunoregulatory programmes in T cells balancing the requirement for a tightly regulated anti‐microbial immune response *versus* initiating an autoimmune reaction.

Our observations concur with previously reported features of the disease and, by sampling patients soon after stroke onset, illustrate the rapid effects of stroke on systemic immunity. As such, alterations to the abundance of monocytes as well as their HLA‐DR expression have been widely reported [31, 69, 84, 85]; however, we demonstrate that it occurs within 3 h of symptom onset. Our study also highlights that the down‐regulation of HLA‐DR is a wider phenomenon, affecting cDC2s and pDCs that play key roles in priming T cell responses and type I interferon production. While myeloid DC precursors have previously been shown to be decreased in circulation following ischaemic stroke [33], in recent years our understanding of DC biology and their associated subsets has grown [86, 87, 88]. Consequently, specific subsets that have been recently identified have not been investigated. In this context, our data outline phenotypical alterations to cDC1s, cDC2s and pDCs in the hyperacute phase post‐stroke. Our data add to mounting evidence that implicate pDCs in the ensuing immune response post‐stroke and illustrate that pDCs could acquire a functionally altered state within hours of stroke, in addition to their role in the sustained immune response post‐stroke [13]. Further, the modest decrease in cDC1 frequencies we observe soon after stroke could result from their recruitment to the ischaemic brain, where they could prime detrimental T cell responses. However, only cDC2s (human: CD1c^+^, mouse: CD172a^+^) have been detected in the brain using post‐mortem brain tissue and following experimental stroke [33, 73]. Regardless, DCs have been shown to be reduced in circulation and exhibit an impaired capacity to secrete cytokines in patients following subarachnoid haemorrhage, a condition that also drives systemic immunosuppression [89]. Emerging evidence has also highlighted the existence of a novel inflammatory subset of cDC2s which are CD5^−^CD163^+^CD14^+^ and has implicated their expansion in systemic lupus erythematosus [90]. Given that we implicate stroke in modulating HLA‐DR expression on cDC2s, it remains to be determined whether this phenotype could be ascribed to the newly described inflammatory cDC2 subset.

Altered monocyte phenotypes have previously been reported in stroke patients [30, 31, 32, 84, 91] and, congruent with previous data, we also identify a decreased expression of HLA‐DR on intermediate monocytes. Although we report no alterations to monocyte proportions in the hyperacute phase of stroke, unlike previous data [31], our sample size and the observed variability could limit our ability to detect these phenotypes. Nevertheless, we identify novel alterations to the expression of CD64 on classical monocytes and CD14 across all monocyte subsets. Enhanced CD14 expression on monocytes has been linked to the acquisition of a tolerance to endotoxin exposure, characterized by an inability to secrete proinflammatory cytokines [62], a phenotype observed in stroke [92]. However, it is unclear if the increased levels of CD14 on monocytes are driven by up‐regulation or the generation and release of CD14^high^ monocytes from the bone marrow, analogous to the concept of innate immune training [15, 93, 94]. Taken together with alterations to CD64 expression, these alterations could probably compromise the ability of patients to response to infectious challenges.

Examining the heterogeneity in immune cells also demonstrated a modest reduction the frequency of HSPCs as well as unswitched B cells in circulation. Studies have shown that in experimental stroke there are alterations in bone marrow haematopoiesis that confers a myeloid bias to HSPCs at the expense of lymphopoiesis, affecting B cell development [17, 95]. Equally, it is plausible that HSPCs could be retained in inflamed tissues such as the ischaemic brain as they circulate and undergo local haematopoiesis [96, 97]. Thus, while we identify a decrease in the frequency of IgD^+^CD27^+^ unswitched memory B cells, it is unclear if alterations in HSPC frequencies could modulate the balance of memory B cell subsets as migration and entry into the germinal centre reaction could occur more rapidly. What is clear, however, is that stroke swiftly alters the dynamics of the circulating memory B cells and potentially humoral responses. Further studies are required to causally implicate the decreased frequencies of unswitched memory B cells in driving the observed hypogammaglobulinaemia post‐stroke [40, 63]. Moreover, it also remains to be determined if stroke directly modulates the frequency and developmental potential of circulating HSPCs.

Our study has several limitations, including a small sample size recruited from a single hospital which might not be representative of the wider population of stroke patients. Serial blood samples in the patients were not obtained for immunophenotyping, meaning that insights into the changes in immune phenotype beyond the hyperacute phase were not possible. Our control and stroke groups are also not precisely matched for co‐morbidities and our data could over‐estimate the effect of stroke on immune function. This is evidenced by recent work that highlighted how risk factors for cerebrovascular disease and their genetic susceptibility loci, e.g. hypertension, obesity, atherosclerosis and hyperlipidaemia, can modulate haematopoiesis and innate immunity [98]. As a result, larger appropriately powered, co‐morbidity‐matched cohorts assessing multiple immune parameters are critical to validate our findings and to determine the relationships with post‐stroke infection and clinical outcomes. Our small sample size and low numbers of patients with infection limit speculation on immunophenotypes that could be predictive of infection, further reinforcing the need for larger studies. Although we profile immune cell subsets, we were unable to concurrently map the systemic cytokine, complement, autonomical and hypothalamic–pituitary adrenal axis response with a similar level of depth due to insufficient blood volumes. Thus, mechanistic studies are essential to determine the differential roles of the adrenergic, cholinergic and glucocorticoid pathways driving early and delayed alterations to immune function. Although we present a snapshot of hyperacute immune alterations, it is equally important to analyse the temporality of immune changes over the acute and post‐acute phases and how they shape outcome following the ischaemic insult; for example, in the context of cognitive decline [13]. Regardless, our study is the first to place an equal emphasis on the innate and adaptive immune compartments and prospectively identifies novel immunophenotypes that track with disease severity during the hyperacute phase of stroke, and that warrant more detailed follow‐up studies.

## Disclosures

The authors declare no competing interests.

## Author contributions

S. H., S. M. A and C. J. S. set up the clinical protocol between UoM and SRFT. C. J. S. and S. H. co‐ordinated the clinical study conduct and blood sampling. S. K., C. B. L, S. M.A. and J. R. G. conceived and designed experiments. S. K. and C. O. performed experiments. S. K. analysed and interpreted data. S. K. wrote and reviewed the paper. All authors edited the paper, provided final approval and vouch for the contents of the final manuscript.

## Data and Availability Statement

All data required to evaluate the conclusions presented in the paper are present in the paper or Supplementary Materials.

## Supporting information


**Fig. S1.** Immune cell types identified in stroke patients. (A,B) Representative FACS plots showing flow cytometric gating strategy employed to identify myeloid cell subsets (**A**) and HSPCs (**B**) in control and stroke patients. Lineage contains CD3, CD19, CD20, CD15, CD56, CD66b.

**Fig. S2.** Composition of the myeloid compartment in patients during the hyper‐acute phase of stroke. (A) Population averaged and normalised gene expression of *CCR2* in the indicated human monocyte and dendritic cell (DC) subsets obtained from the Human Cell Atlas project. (**B‐D**) Frequencies of granulocytes (**B**), monocytes and their subsets (**C**) and DCs (**D**) amongst live cells in controls (*n* = 13) and stroke patients (*n* = 11). (**E,F**) Correlation plot of cDC1 frequencies with stroke severity (NIHSS) (**E**) and HLA‐DR expression on cDC2s with NIHSS and age (**F**). Data are presented as bars showing median values and dots representing individual data points. In correlations, the regression line and standard error are shown. Statistical comparisons were performed using a Mann Whitney *U* test and correlations using Spearman ranked coefficient correlation test.

**Fig. S3.** The T cell compartment in patients during the hyper‐acute phase of stroke. (A) Frequencies of CD4^+^, CD8^+^, Vδ2^+^ and other γδ T cells amongst CD3^+^ T cells in controls (*n* = 12) and stroke patients (*n* = 10). (**B**) Frequencies of naïve, TCM, TEM and TEMRA CD8^+^ T cell subsets amongst CD8^+^ T cells in controls (*n* = 12) and stroke patients (*n* = 10). (**C**) Correlation plots of CD69^+^ naïve and TCM CD4^+^ cells with stroke severity (NIHSS). Data are presented as bars showing median values and dots representing individual data points. In correlations, the regression line and standard error are shown. Statistical comparisons were performed using a Mann Whitney *U* test and correlations using Spearman ranked coefficient correlation test.
