# Supplementary figures and images for: A hyperacute immune map of ischaemic stroke patients reveals alterations to circulating innate and adaptive cells
Source: Clin Exp Immunol. 2020 Dec 9;203(3):458–71. doi: 10.1111/cei.13551 (PMC7874838; doi:10.1111/cei.13551)

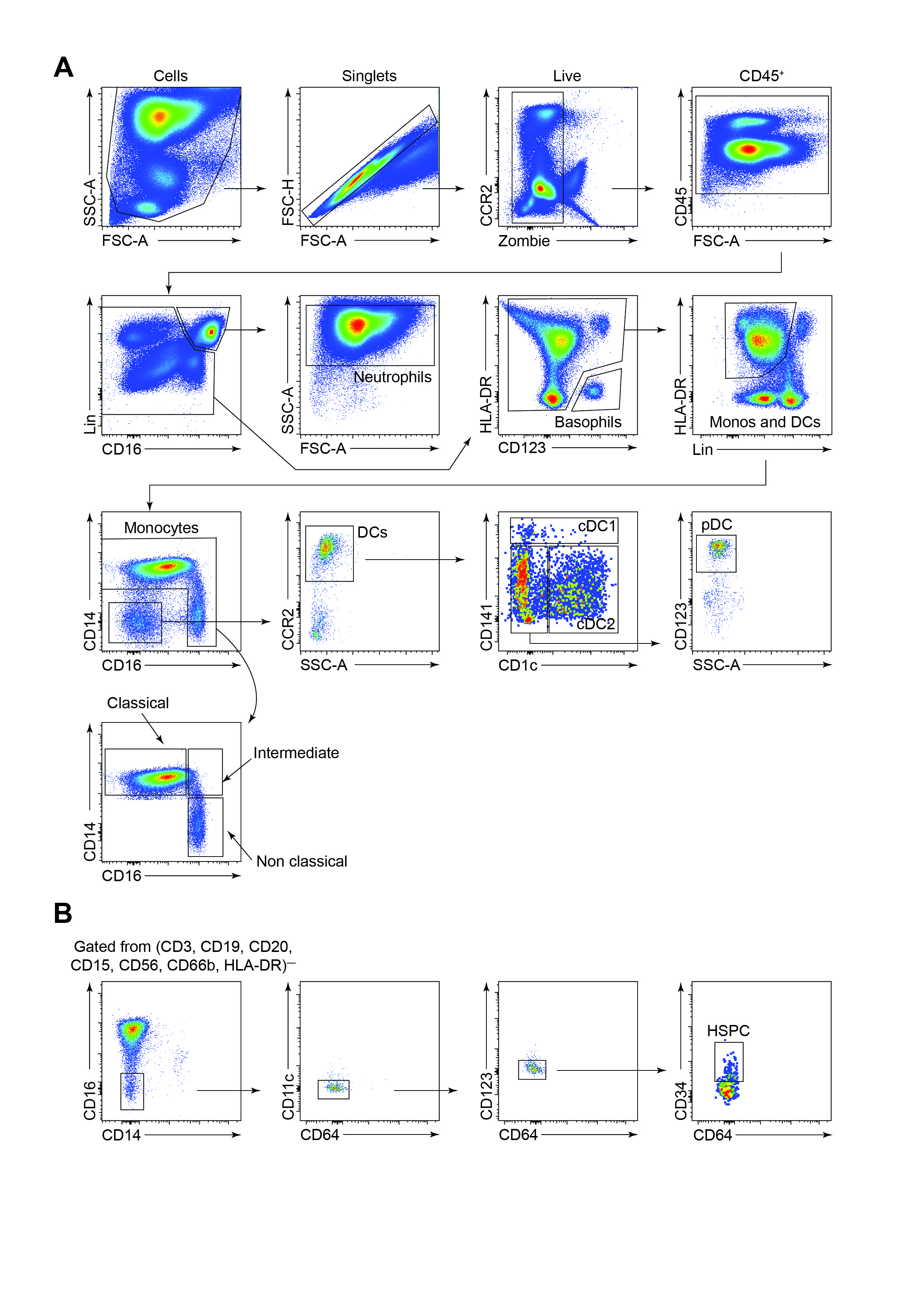

Supplement: Supplementary file 1 — Fig. S1. Immune cell types identified in stroke patients. (A,B) Representative FACS plots showing flow cytometric gating strategy employed to identify myeloid cell subsets (A) and HSPCs (B) in control and stroke patients. Lineage contains CD3, CD19, CD20, CD15, CD56, CD66b. [file CEI-203-458-s001.jpg]

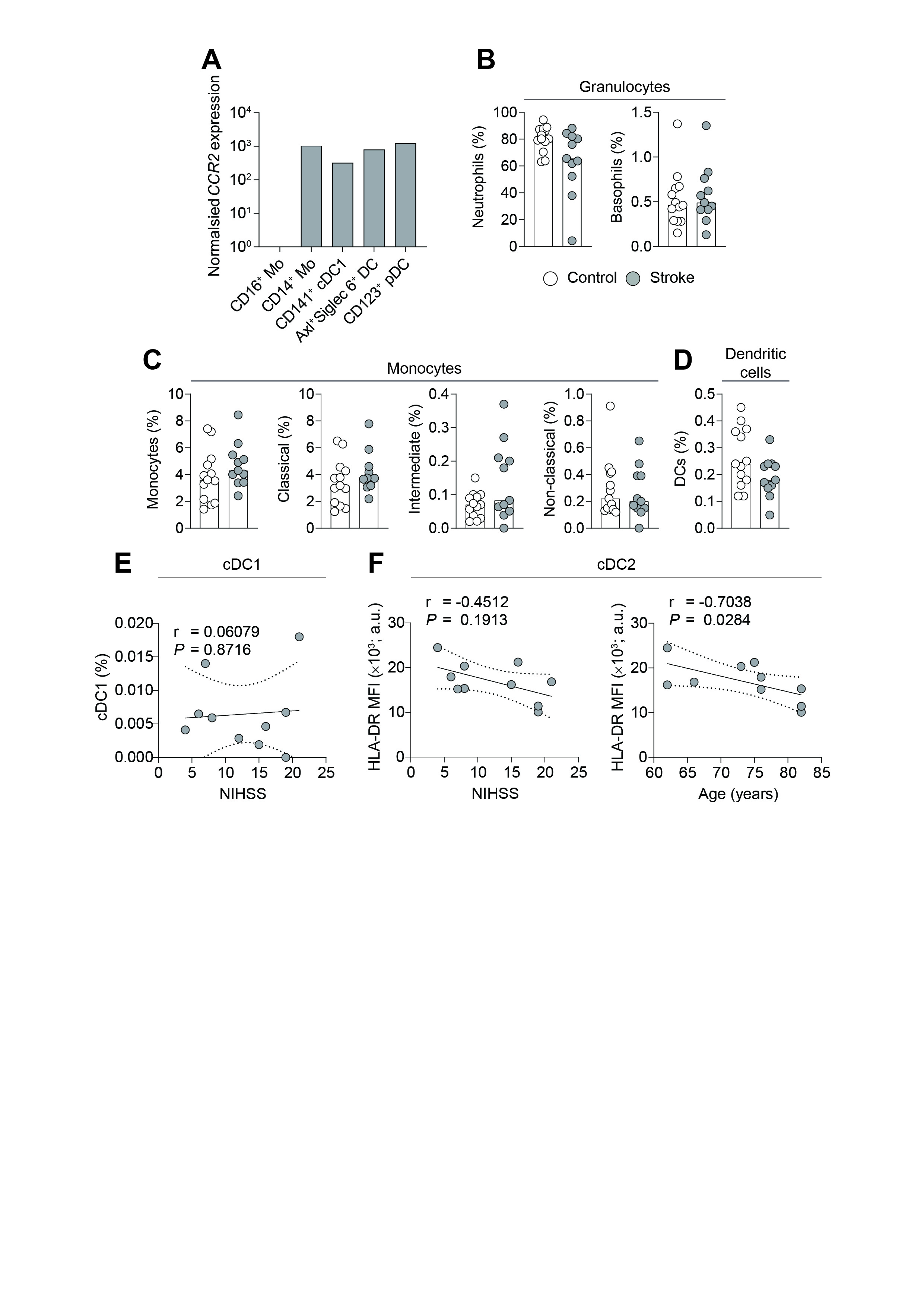

Supplement: Supplementary file 2 — Fig. S2. Composition of the myeloid compartment in patients during the hyper‐acute phase of stroke. (A) Population averaged and normalised gene expression of CCR2 in the indicated human monocyte and dendritic cell (DC) subsets obtained from the Human Cell Atlas project. (B‐D) Frequencies of granulocytes (B), monocytes and their subsets (C) and DCs (D) amongst live cells in controls (n = 13) and stroke patients (n = 11). (E,F) Correlation plot of cDC1 frequencies with stroke severity (NIHSS) (E) and HLA‐DR expression on cDC2s with NIHSS and age (F). Data are presented as bars showing median values and dots representing individual data points. In correlations, the regression line and standard error are shown. Statistical comparisons were performed using a Mann Whitney U test and correlations using Spearman ranked coefficient correlation test. [file CEI-203-458-s002.jpg]

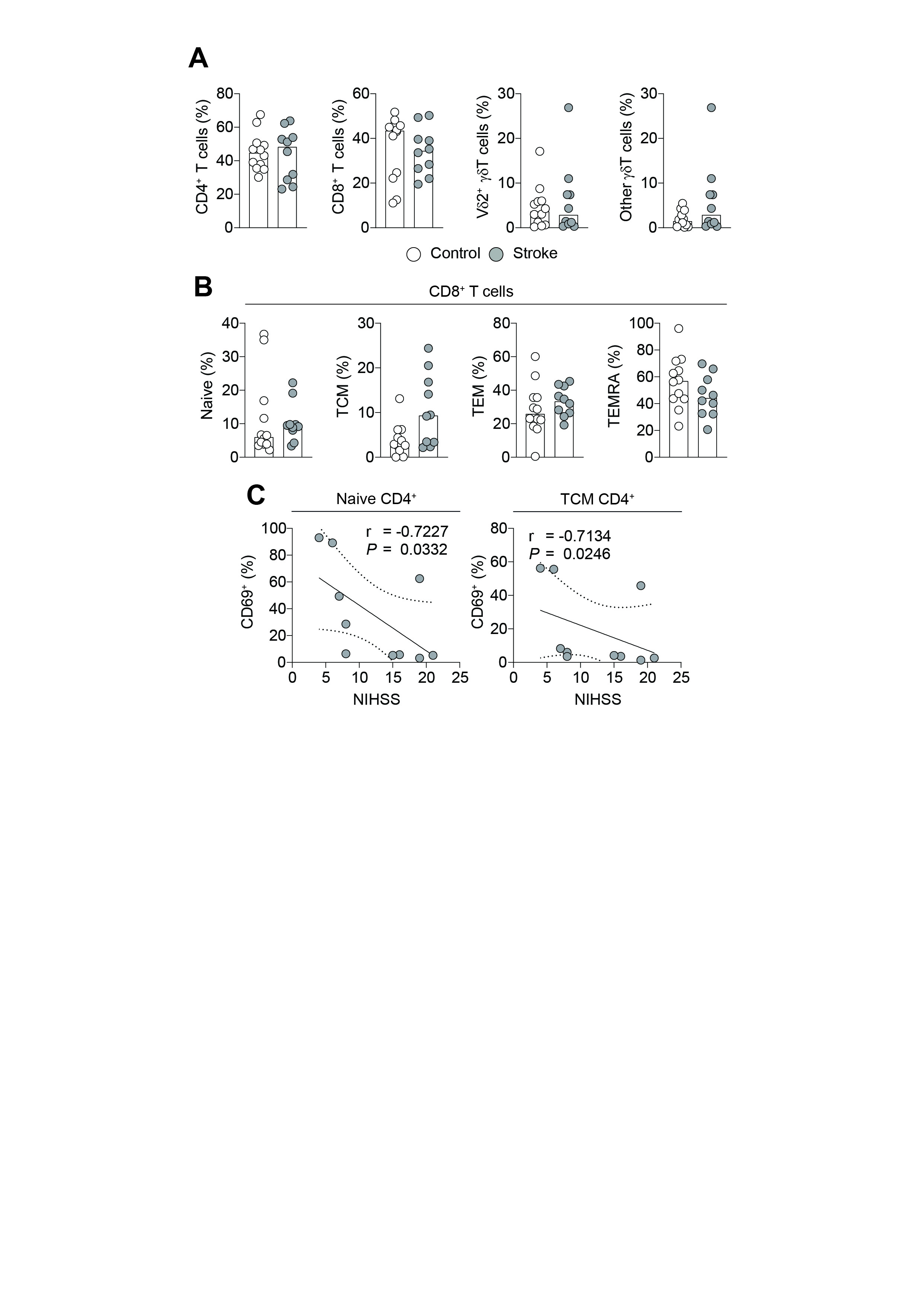

Supplement: Supplementary file 3 — Fig. S3. The T cell compartment in patients during the hyper‐acute phase of stroke. (A) Frequencies of CD4+, CD8+, Vδ2+ and other γδ T cells amongst CD3+ T cells in controls (n = 12) and stroke patients (n = 10). (B) Frequencies of naïve, TCM, TEM and TEMRA CD8+ T cell subsets amongst CD8+ T cells in controls (n = 12) and stroke patients (n = 10). (C) Correlation plots of CD69+ naïve and TCM CD4+ cells with stroke severity (NIHSS). Data are presented as bars showing median values and dots representing individual data points. In correlations, the regression line and standard error are shown. Statistical comparisons were performed using a Mann Whitney U test and correlations using Spearman ranked coefficient correlation test. [file CEI-203-458-s003.jpg]
